# Optimizing isolated limb perfusion in malignant melanoma and sarcoma: Key factors influencing postoperative outcomes and complications

**DOI:** 10.1097/MD.0000000000050929

**Published:** 2026-09-25

**Authors:** Veysel Cem Özcan, Ezgi Altinsoy, Abdurrahman Başpinar, İsmail Can Tercan, Firat Tekeş, Selim Tamam, Serdar Çulcu, Ali Ekrem Ünal

**Affiliations:** a Surgical Oncology Department, Antalya City Hospital, Antalya, Türkiye; b Surgical Oncology Department, Van Training and Research Hospital, Van, Türkiye; c Surgical Oncology Department, Ordu University Training and Research Hospital, Ordu, Türkiye; d Surgical Oncology Department, Diyarbakir Gazi Yaşargil Training and Research Hospital, Diyarbakir, Türkiye; e Surgical Oncology Department, Ankara Etlik City Hospital, Ankara, Türkiye; f Surgical Oncology Department, Ankara University Faculty of Medicine, Ankara, Türkiye.

**Keywords:** Clavien-Dindo, isolated limb perfusion, malignant melanoma, soft tissue sarcoma, Wieberdink

## Abstract

Isolated hyperthermic limb perfusion (ILP) is a limb-salvage technique used in the treatment of malignant melanoma and soft tissue sarcomas with in-transit or locally advanced disease. ILP allows regional administration of high-dose chemotherapy with minimal systemic toxicity. This study aims to evaluate postoperative complications and identify predictive factors influencing outcomes in patients undergoing ILP. We retrospectively analyzed 49 patients with malignant melanoma or extremity soft tissue sarcoma who underwent ILP at a single tertiary center between 2014 and 2024. Complications were classified using the Clavien-Dindo system; toxicity was graded via the Wieberdink scale. Logistic regression and univariate analyses were used to evaluate clinical, surgical, and laboratory parameters predictive of severe complications, toxicity, and mortality. Of the patients, 32 (65.3%) experienced complications, primarily within the first 3 postoperative days. Severe complications (Clavien-Dindo grade ≥ 3) occurred in 11 patients (22.4%). Severe regional toxicity (Wieberdink grade ≥ 4) was observed in 6.1%. Early mortality occurred in 3 cases. Preoperative and postoperative albumin levels, along with C-reactive protein levels on postoperative days 3 and 5, were significantly associated with severe complications. No significant relationship was found between modified Systemic Inflammation Scores and postoperative outcomes. ILP remains a valuable regional treatment option for carefully selected patients with advanced melanoma and extremity soft tissue sarcoma. Preoperative nutritional status and early postoperative inflammatory markers, particularly albumin and C-reactive protein, may serve as valuable predictors for adverse outcomes. Optimization of these parameters could improve postoperative recovery and treatment planning.

## 1. Introduction

Isolated hyperthermic limb perfusion (ILP) was first introduced by Creech et al in 1957 as a novel approach for the treatment of sarcoma and malignant melanoma.^[1]^

Melanomas are malignant tumors originating from melanocytes. Melanocytes produce melanin pigment and are primarily found in the skin; however, they can also be present in the eyes, ears, leptomeninges, gastrointestinal tract, and oral, genital, and sinonasal mucosal membranes. Malignant melanoma predominantly affects the white population, with cutaneous melanomas constituting the majority of cases (over 90%), while mucosal and uveal melanomas are much rarer (<1%–5%, with country-specific variations).^[2]^

The incidence is 25 new cases per 100,000 people in Europe, 30 in the United States, and 60 in Australia and New Zealand annually.^[3]^ Approximately 10% of cutaneous melanoma cases present with locoregional metastases at the time of diagnosis, such as transit and satellite metastases, as well as regional lymph node metastases.^[4]^ According to current guidelines, ILP is indicated for patients with malignant melanoma in cases of transit metastases, satellitosis, localized cutaneous or soft tissue metastases restricted to a limb, and when tumor control cannot be achieved by other methods.^[5]^

Soft tissue sarcomas (STS) represent a heterogeneous group of diseases characterized by numerous histological subtypes, constituting <1% of all malignancies in adults.^[6]^ STS can develop in any anatomical region, with approximately 60% occurring in the limbs. In patients with extremity soft tissue sarcomas (ESTS), the key determinant of survival is the presence of metastasis; therefore, local tumor treatment is of secondary importance. Consequently, local treatment of these tumors has evolved from amputation to limb-sparing surgery combined with radiotherapy.^[7]^ For unresectable, locally advanced, or recurrent STS, limb-sparing treatments include ILP with tumor necrosis factor alpha (TNF-α) and melphalan. In patients with ESTS, the success rate of ILP in limb salvage approaches 80%.^[8,9]^

One of the advantages of ILP is the potential to administer high-dose cytotoxic chemotherapy along with TNF-α, a vasoactive cytokine, while minimizing the risk of severe systemic toxicity. The dose of the cytotoxic drug is determined by the volume of the limb under the tourniquet, which depends on numerous factors such as age, weight, nutritional status, and tumor burden.^[10,11]^

Initially, doxorubicin was used as the chemotherapeutic agent, but it was later found that melphalan provided superior efficacy and lower toxicity compared with doxorubicin and other agents. Due to its short half-life and low relative toxicity to blood vessels and soft tissues, melphalan is considered the most suitable chemotherapeutic agent for ILP.

TNF-α, a natural mediator of the systemic inflammatory response, has both direct and indirect tumoricidal effects. In addition, TNF-α negatively impacts tumor neovascularization, enhancing the permeability of tissues and facilitating better diffusion of melphalan into the tumor tissue.^[12]^

As part of the systemic inflammatory response to the tumor, pro-inflammatory cytokines and growth factors are released, exerting a catabolic effect on host metabolism. Interleukin-6, produced by the tumor or surrounding cells, stimulates the liver to produce acute-phase proteins (such as C-reactive protein [CRP], fibrinogen, etc) both in the fasting and fed states. It may also regulate the synthesis of albumin by hepatocytes. In the early stages of cancer, mild hypoalbuminemia may be observed, or albumin levels may remain normal, but as the disease progresses, albumin levels decrease significantly, and this decrease can be crucial in determining cancer prognosis.^[13]^

Recent studies have identified measurable parameters of the systemic inflammatory response as independent prognostic markers for various cancer types. Research has shown that in certain cancers, leukocytosis, thrombocytosis, a high neutrophil-to-lymphocyte ratio, and platelet-to-lymphocyte ratio are significantly associated with poor survival.^[14–19]^

In this study, we aim to present the postoperative outcomes of patients with malignant melanoma and STS who underwent ILP and investigate the parameters that may influence postoperative complications.

## 2. Materials and methods

This study investigated patients who underwent ILP at the Surgical Oncology Department of Ankara University Faculty of Medicine between 2014 and 2024. The patient group consisted of individuals followed up for malignant melanoma and ESTS. The study was designed retrospectively. Factors influencing the development of complications and toxicity in patients who underwent ILP were evaluated. Complications were assessed according to the Clavien-Dindo classification, while toxicity was evaluated using the Wieberdink classification. The modified Systemic Inflammation Score (mSIS) was calculated based on serum albumin levels and the lymphocyte-to-monocyte ratio (LMR), as previously described by Morkavuk et al.^[20]^ Patients with albumin ≥4.0 g/dL and LMR ≥3.4 were classified as mSIS 0; patients with either albumin <4.0 g/dL or LMR <3.4 were classified as mSIS 1; and patients with both albumin <4.0 g/dL and LMR <3.4 were classified as mSIS 2. The mSIS was calculated preoperatively and on postoperative days 1, 3, and 5. Ethical approval for the study was obtained from the Ankara University Ethics Committee under the application number 2024000149-4.

### 2.1. Patient selection

Patients who underwent ILP for malignant melanoma and ESTS were included in the study. To minimize selection bias, all consecutive eligible patients who underwent isolated limb perfusion during the study period were included in the analysis. There were no age restrictions. In patients with malignant melanoma, ILP was performed for those with in-transit metastases and bulky lymph nodes. In patients with sarcoma, ILP was applied before definitive surgery to induce tumor regression for limb-sparing purposes. All patients who met the indications were included in the study, and no patients were excluded.

### 2.2. Surgical technique

For tumors located in the lower extremity, including the thigh and distal regions, the femoral artery and vein were used, while for tumors in the inguinal region, the iliac artery and vein were utilized. Access to the iliac artery and vein was achieved through a retroperitoneal approach. In cases of upper extremity lesions, the axillary and subclavian arteries were used. The arteriovenous structures were suspended, and 5000 IU of heparin was administered as a bolus before clamping the distal and proximal vessels. After a 3-minute wait post-heparinization, arteriotomy and venotomy were performed. Subsequently, catheters of appropriate size were inserted into the artery and vein.

Melphalan was used as the chemotherapeutic agent. The volume of the limbs was calculated, and the perfusion concentration was determined to be 10 mg/L for the upper extremity and 12 mg/L for the lower extremity. A combination of melphalan and fluid was infused into the limbs at 40°C for 60 minutes. In addition, during the procedure, oxygen was infused into the extremities via the arterial cannula at a rate of 2 to 4 L/min. To prevent systemic toxicity, the perfusate was passed through a hemofilter for 15 minutes at the end of the perfusion. After the procedure, the cannulas were removed, and the arteriotomy and venotomy incisions were sutured. Pulse control of the extremity was performed through physical examination and a portable Doppler device.

### 2.3. Statistical analysis

Descriptive statistics are presented as frequency, percentage, and median (first quartile−third quartile). The normality of the variables was assessed using the Shapiro–Wilk test, and it was determined that they were not normally distributed. The Pearson chi-square test was applied for comparing categorical variables in independent groups. The Mann–Whitney *U* test was used for comparing continuous variables between 2 groups. Logistic regression analysis with the backward model was performed to predict severe toxicity and serious complications. A significance level of *P* < .05 was considered statistically significant in the study. Statistical analyses of the research data were performed using the Statistical Package for Social Sciences version 25.0 for Windows (IBM Corp., Armonk). No formal sample size calculation was performed because all eligible consecutive patients treated during the study period were included.

### 2.4. Competing interests

The authors declare that they have no competing interests.

## 3. Results

A total of 49 patients were included in the study. The patients underwent ILP due to malignant melanoma or extremity sarcoma. The demographic characteristics of the patients are shown in Table 1. Of the patients, 16 were female (32.7%), and 33 were male (67.3%). The mean age of the patients was 49.4 years (range: 11–86 years), and the median age was 51 years.

**Table 1:** Patient demographics.

	n (%)
Gender	
Female	16 (32.7)
Male	33 (67.3)
Comorbidity	
No	32 (65.3)
Yes	17 (34.7)
Chronic illness	
Diabetes mellitus	4 (8.2)
Chronic lymphocytic leukemia	1 (2)
Hypertension	5 (10.2)
Asthma	1 (2)
Coronary artery disease	2 (4.1)
Hypothyroidism	2 (4.1)
Breast cancer	1 (2)
Hyperlipidemia	1 (2)
ASA score	
1	8 (16.3)
2	25 (51)
3	16 (32.7)
4	0 (0)
5	0 (0)
ASA group	
ASA 1-2	33 (67.3)
ASA 3-4-5	16 (32.7)
BMI	
Underweight	5 (10.2)
Normal weight	18 (36.7)
Overweight	20 (40.8)
Obese	6 (12.2)
Morbidly obese	0 (0)

ASA = American Society of Anesthesiologists, BMI = body mass index.

In 40 patients, the tumor was located in the lower extremity, while in 9 patients, the tumor was located in the upper extremity. In the procedures for the upper extremity, the axillary and subclavian arteries were used, while for the lower extremity, the iliac or femoral arteries were utilized depending on the location of the lesion. ILP was performed for malignant melanoma in 31 patients (63.3%) and for sarcoma in 18 patients (36.7%; Table 2).

**Table 2:** Postoperative results.

	n (%)
Tumor location	
Lower extremity	40 (81.6)
Upper extremity	9 (18.4)
Vessel used for perfusion	
Axillary artery	5 (10.2)
Subclavian artery	4 (8.2)
Iliac artery	26 (53)
Femoral artery	14 (28.6)
Previous surgery	
None	17 (34.7)
Excision	29 (59.2)
Amputation	3 (6.1)
Diagnosis	
Melanoma	31 (63.3)
Sarcoma	18 (36.7)
Adjuvant therapy	
No	24 (49)
Yes	25 (51)
Radiotherapy	
No	35 (71.4)
Yes	14 (28.6)
Chemotherapeutic agent	
Melphalan	37 (75.5)
Melphalan + TNF-alpha	4 (8.2)
Doxorubicin	6 (12.2)
Doxorubicin + TNF-alpha	2 (4.1)
Primary or recurrent	
Primary	28 (57.1)
Recurrent	21 (42.9)
Intraoperative transfusion	
No	47 (95.9)
Yes	2 (4.1)
Postoperative transfusion	
No	34 (69.4)
Yes	15 (30.6)
Number of extracted lymph nodes	
0	7 (14.3)
1	7 (14.3)
2	7 (14.3)
3	1 (2)
4	3 (6.1)
5	6 (12.2)
6	4 (8.2)
7	2 (4.1)
8	3 (6.1)
9	2 (4.1)
14	2 (4.1)
17	2 (4.1)
20	1 (2)
22	1 (2)
26	1 (2)

TNF-α = tumor necrosis factor alpha.

Seventeen patients (34.7%) did not develop any complications, while 32 patients (65.3%) experienced complications. Among those with complications, it was observed that 71.9% of complications occurred within the first 3 days postoperatively. Of the 32 patients who developed postoperative complications, 23 (71.9%) experienced their first complication within the initial 72 hours after ILP. The most frequent early complications were surgical site infection (n = 10) and seroma/edema (n = 8), whereas acute respiratory distress syndrome (n = 2), hemorrhage (n = 2), and neurological injury (n = 1) were less common. The majority of all complications were wound infections and seromas (55.1%).

The Clavien-Dindo complication, Wieberdink toxicity, and mortality rates of the patients were assessed. To analyze the factors influencing these parameters, the Clavien-Dindo complication groups were divided into mild and severe complications, where grades 1 and 2 were considered mild complications and grades 3, 4, and 5 were classified as severe complications. In the Wieberdink scale, grades 1 and 2 were considered mild toxicity, and grades 3, 4, and 5 were classified as severe toxicity. According to this classification, 38 patients (77.6%) had mild complications, while 11 patients (22.4%) had severe complications.

In the evaluation of toxicity, 46 patients (93.9%) experienced mild toxicity according to the Wieberdink scale, while 3 patients (6.1%) experienced severe toxicity.

Early postoperative mortality was also examined. Three patients died during the early postoperative period. In the general population, it was found that 22 of the 49 patients were still alive, while 27 patients had died (Table 3).

**Table 3:** Postoperative complications.

	n (%)
Complication	
None	17 (34.7)
Surgical site infection	18 (36.7)
Seroma/edema	9 (18.4)
ARDS	2 (4.1)
Neurological injury	1 (2)
Hemorrhage	2 (4.1)
Complication time (d)	
1	6 (12.2)
2	8 (16.3)
3	9 (18.4)
4	3 (6.1)
5	5 (10.2)
9	1 (2)
Wieberdink toxicity	
1	23 (46.9)
2	13 (26.5)
3	10 (20.4)
4	3 (6.1)
5	0 (0)
Wieberdink group	
Mild toxicity	46 (93.9)
Severe toxicity	3 (6.1)
Clavien-Dindo complication	
1	15 (30.6)
2	23 (46.9)
3	9 (18.4)
4	1 (2)
5	1 (2)
Clavien-Dindo group	
Mild complication	38 (77.6)
Severe complication	11 (22.4)
Early mortality	
No	46 (93.9)
Yes	3 (6.1)
Mortality	
No	22 (44.9)
Yes	27 (55.1)
Mortality cause	
None	22 (44.9)
Metastasis	20 (40.8)
Sepsis	5 (10.2)
Toxic hepatitis	1 (2)
Cardiac arrest	1 (2)

ARDS = acute respiratory distress syndrome.

Prognostic markers that might influence complications, toxicity, and mortality were further investigated. The relationship between complications, toxicity, and mortality was analyzed with respect to demographic data, preoperative and postoperative laboratory findings, whether or not transfusions were administered, and surgical techniques (Table 4).

**Table 4:** Analysis of factors affecting complications.

Variables	Mild complication (n = 38)	Severe complication (n = 11)	*P* value
Age	47 (33–64)	57 (50.5–60)	.297
NLR			
Preoperative NLR	4.4 (2.7–6.5)	4.5 (3.8–8.5)	.741
Postoperative day 1 NLR	9.7 (5.8–14.7)	7.6 (6.8–16)	.719
Postoperative day 3 NLR	6.1 (4.1–10.1)	6.5 (5.8–7.2)	.508
Postoperative day 5 NLR	5.9 (3.7–9.2)	7.8 (4.2–13.3)	.408
LMR			
Preoperative LMR	2.7 (1.5–4.5)	2.6 (1.2–3.9)	.388
Postoperative day 1 LMR	1.4 (1–2.2)	1.7 (1.2–2.2)	.461
Postoperative day 3 LMR	2.9 (2–4.9)	2.3 (2–3)	.144
Postoperative day 5 LMR	4.6 (2.5–7.4)	3.6 (2.8–7.1)	.825
Albumin (g/L)			
Preoperative albumin	40.3 (35.3–45.1)	27.5 (22.8–37.5)	**.004**
Postoperative day 1 albumin	31.4 (28.8–33.6)	23.4 (20.5–32.8)	.069
Postoperative day 3 albumin	31.3 (28.6–35)	25.6 (22.9–31.9)	**.044**
Postoperative day 5 albumin	33.6 (30.8–40)	27.7 (24.9–30.9)	**.016**
ASA score	2 (2–3)	2 (2–3)	.269
BMI	25.1 (23.5–28.4)	24.6 (18.5–25.3)	.161
CRP (mg/L)			
Preoperative CRP	8.3 (1.6–31.2)	34.5 (9.9–81.1)	.065
Postoperative day 1 CRP	38.2 (18.9–71.4)	66 (23.4–140.5)	.108
Postoperative day 3 CRP	70.1 (30.9–117)	103.3 (70.1–194)	**.049**
Postoperative day 5 CRP	53.5 (22–99.5)	100 (57.6–167.2)	**.036**
mSIS			
Preoperative mSIS	0 (0–2)	2 (1–2)	.054
Postoperative day 1 mSIS	2 (2–2)	2 (2–2)	.432
Postoperative day 3 mSIS	2 (1–2)	2 (2–2)	.170
Postoperative day 5 mSIS	1 (0–1)	1 (1–1.5)	.568
Intraoperative transfusion			
No	37 (97.4%)	10 (90.9%)	.402
Yes	1 (2.6%)	1 (9.1%)	
Surgical technique			
Excision + Perfusion	9 (23.7%)	2 (18.2%)	.700
Perfusion	29 (76.3%)	9 (81.8%)	
Comorbidity			
No	26 (68.4%)	6 (54.5%)	.480
Yes	12 (31.6%)	5 (45.5%)	

Bold values indicate statistical significance (*P* < .05).

ASA = American Society of Anesthesiologists, BMI = body mass index, CRP = C-reactive protein, LMR = lymphocyte-to-monocyte ratio, mSIS = modified Systemic Inflammation Score, NLR = neutrophil-to-lymphocyte ratio.

Univariate analysis showed that preoperative and postoperative albumin levels on the third and fifth days, as well as CRP values on the third and fifth days postoperatively, were effective parameters in predicting severe complications according to the Clavien-Dindo classification.

When analyzing parameters that could predict severe toxicity according to the Wieberdink toxicity scale, no statistically significant prognostic marker was identified (Table 5).

**Table 5:** Analysis of factors affecting toxicity.

Variables	Mild toxicity (n = 46)	Severe toxicity (n = 3)	*P* value
Age	50.5 (33–63)	59 (53.5–72)	.196
NLR			
Preoperative NLR	4.42 (2.7–6.6)	4.445 (4.38–4.51)	.959
Postoperative day 1 NLR	9.895 (6.1–15.3)	7.48 (6.34–7.54)	.278
Postoperative day 3 NLR	6.4 (4.4–10.1)	6.3 (5.8–6.38)	.815
Postoperative day 5 NLR	5.935 (3.65–9.3)	7.75 (5.875–10.175)	.559
LMR			
Preoperative LMR	2.485 (1.44–4.4)	3.14 (2.125–3.43)	.707
Postoperative day 1 LMR	1.41 (1–2.15)	4.02 (1.98–6.06)	.134
Postoperative day 3 LMR	2.765 (1.96–4.48)	2.3 (2.26–3.2)	.868
Postoperative day 5 LMR	4.39 (2.5–7)	7.1 (5.285–7.8)	.317
Albumin (g/L)			
Preoperative albumin	39.9 (34–44.2)	25.1 (20.45–32)	.066
Postoperative day 1 albumin	31.1 (27–33.6)	23.4 (19.8–28.15)	.233
Postoperative day 3 albumin	31.1 (26–35)	22.7 (22.4–26.3)	.053
Postoperative day 5 albumin	32.5 (28–39.9)	24.7 (24.55–27.75)	.058
ASA score	2 (2–3)	3 (2.5–3)	.184
BMI	25 (23.4–28.4)	24.6 (21.05–29.5)	1.000
CRP (mg/L)			
Preoperative CRP	11.2 (4.6–38.3)	34.5 (20.75–59.15)	.317
Postoperative day 1 CRP	40.75 (18.9–89)	61.6 (40.9–113.3)	.453
Postoperative day 3 CRP	78.95 (32–136.3)	86.7 (66.1–145.85)	.505
Postoperative day 5 CRP	61.2 (28.05–106)	54.2 (53–127.6)	.601
Intraoperative transfusion			
No	44 (95.7%)	3 (100%)	1.000
Yes	2 (4.3%)	0 (0%)	
Surgical technique			
Excision + perfusion	11 (23.9%)	0 (0%)	.580
Perfusion	35 (76.1%)	3 (100%)	
Clavien-Dindo group			
Mild complication	38 (82.6%)	0 (0%)	**.009**
Severe complication	8 (17.4%)	3 (100%)	
mSIS			
Preoperative mSIS	0.5 (0–2)	2 (1.5–2)	.349
Postoperative day 1 mSIS	2 (2–2)	2 (1.5–2)	.338
Postoperative day 3 mSIS	2 (1–2)	2 (1.5–2)	.906
Postoperative day 5 mSIS	1 (1–2)	1 (1–1)	.871

Bold value indicates statistical significance (*P* < .05).

ASA = American Society of Anesthesiologists, BMI = body mass index, CRP = C-reactive protein, LMR = lymphocyte-to-monocyte ratio, mSIS = modified Systemic Inflammation Score, NLR = neutrophil-to-lymphocyte ratio.

Similarly, no statistically significant marker was found when analyzing factors associated with early postoperative mortality. Factors predicting overall survival were also examined, and a statistically significant relationship was found between preoperative neutrophil-to-lymphocyte ratio, preoperative and postoperative albumin levels, and preoperative and postoperative CRP values on the fifth day and overall mortality (Table 6).

**Table 6:** Analysis of factors affecting mortality.

Variables	Live (n = 22)	Exitus (n = 27)	*P* value
Age	42.5 (34–59.5)	53 (45–63)	.295
NLR			
Preoperative NLR	3.7 (2.4–5.2)	5 (3.2–8.9)	**.044**
Postoperative day 1 NLR	10.2 (6.4–16.3)	9.6 (6.1–14.4)	.714
Postoperative day 3 NLR	5.9 (4.3–7.1)	6.4 (5–11.2)	.343
Postoperative day 5 NLR	6.3 (3.7–8.7)	6 (3.9–11.6)	.452
LMR			
Preoperative LMR	3.4 (1.9–4.6)	2.1 (1.3–4)	.272
Postoperative day 1 LMR	1.5 (1–2.1)	1.5 (1–2.2)	.975
Postoperative day 3 LMR	3.1 (2.1–4.7)	2.7 (1.8–3.7)	.276
Postoperative day 5 LMR	4.4 (2.9–6.1)	5.5 (2.3–7.4)	.849
Albumin (g/L)			
Preoperative albumin	43.7 (39.4–45.8)	35 (27.5–40.1)	**<.001**
Postoperative day 1 albumin	32.1 (30.2–34.9)	29.2 (21.4–33)	**.030**
Postoperative day 3 albumin	33 (30.1–36.5)	30 (23.7–33)	**.018**
Postoperative day 5 albumin	39.9 (32.5–41)	30.8 (25.1–34)	**<.001**
ASA score	2 (1.5–2)	2 (2–3)	**.024**
BMI	25.1 (23.8–28.5)	24.6 (22.2–27.1)	.299
CRP (mg/L)			
Preoperative CRP	6.9 (1.2–18.7)	17 (5.7–54)	**.041**
Postoperative day 1 CRP	32.6 (12–74.3)	54.3 (22.5–104.1)	.099
Postoperative day 3 CRP	55 (21.5–108.9)	94 (44.1–147.8)	.102
Postoperative day 5 CRP	49.4 (18.1–74.2)	65.1 (37.7–180.4)	**.033**
Intraoperative transfusion			
No	22 (100%)	25 (92.6%)	.507
Yes	0 (0%)	2 (7.4%)	

Bold values indicate statistical significance (*P* < .05).

ASA = American Society of Anesthesiologists, BMI = body mass index, CRP = C-reactive protein, LMR = lymphocyte-to-monocyte ratio, NLR = neutrophil-to-lymphocyte ratio.

Multivariable logistic regression analysis identified preoperative albumin as an independent predictor of severe postoperative complications (odds ratio = 0.854, 95% confidence interval = 0.770–0.947, *P* = .003). Similarly, postoperative day 5 albumin remained independently associated with severe complications (odds ratio = 0.881, 95% confidence interval = 0.780–0.995, *P* = .041), indicating that lower albumin levels were associated with an increased risk of severe postoperative morbidity.

No statistically significant relationship was found between mSIS values and complications, toxicity, or mortality.

Furthermore, no statistically significant relationship was observed between the number of lymph nodes excised and the formation of seromas or edema.

## 4. Discussion

In this study, we focused on complications and prognostic markers in patients undergoing ILP for malignant melanoma and sarcoma. Our key finding was that preoperative and postoperative albumin levels, along with postoperative CRP levels, were the most significant markers for predicting complications. The multivariable analysis further strengthened these findings by demonstrating that both preoperative and postoperative day 5 albumin independently predicted severe complications, supporting the role of albumin as a robust perioperative biomarker rather than merely an inflammatory surrogate.

Interestingly, we did not find a statistically significant association between mSIS values and complications, which is in contrast to previous research where systemic inflammatory scores were associated with worse outcomes.^[20]^ However, preoperative mSIS showed a trend toward an association with severe complications (*P* = .054), although it did not reach statistical significance. One possible explanation is the different composition of our study population. While previous studies predominantly evaluated melanoma patients, our cohort also included a substantial proportion of patients with ESTS. Given the biological heterogeneity of STS, including differences in tumor biology, vascular invasion, and host inflammatory response, systemic inflammation-based scores such as mSIS may have lower discriminatory performance in a mixed melanoma–sarcoma population. In addition, the relatively small sample size may have limited our ability to detect more subtle associations.

ILP has been widely recognized in the literature as a safe and effective method for treating advanced melanoma and sarcoma, particularly when conventional treatments are insufficient. However, as with any invasive procedure, complications remain a concern. In our study, the rate of severe regional toxicity (Wieberdink 4–5) was found to be 6.1%. This is consistent with previous findings where toxicity rates ranged from 0% to 7% (median of 2.5%) in a meta-analysis including 2637 patients in 2021.^[21]^ These figures suggest that while ILP is generally safe, there remains a risk of significant adverse effects, especially for patients with larger tumor burdens or advanced disease.

Clavien-Dindo complication grading in our study showed that 9 patients (18.4%) experienced grade 3 complications, while 1 patient (2%) experienced a grade 4 complication and another (2%) experienced a grade 5 complication, resulting in an overall severe complication rate of 22.4%. Although grade 4–5 complications were uncommon and comparable with the low rates of major complications reported in previous studies,^[22,23]^ the inclusion of grade 3 events resulted in a higher overall rate of severe complications in our cohort. It is also important to note that previous studies investigating toxicity and complications have predominantly included melanoma patients. Given the nature of sarcomas, which may involve more extensive local and vascular invasion and aggressive tumor characteristics, the inclusion of a substantial proportion of sarcoma patients in our cohort may have contributed to the observed higher overall complication rate.

A particularly notable finding in our study is the early onset of complications, with 72% of complications occurring within the first 3 days postoperatively. This aligns with the findings of other studies where the majority of complications following ILP were reported early in the postoperative period.^[24]^ Because most complications became clinically evident within the first 72 hours, the concomitant decline in albumin and increase in CRP observed during this early postoperative period supports the use of serial biomarker monitoring for early identification of patients at higher risk of severe postoperative complications. Although our study was not designed to establish a temporal or causal relationship between individual complications and biomarker fluctuations, these findings suggest that routine monitoring of albumin and CRP may facilitate earlier clinical assessment and timely intervention.

Through univariate analyses, we identified that preoperative, postoperative day 3, and postoperative day 5 albumin levels, as well as CRP levels on postoperative days 3 and 5, were predictive of severe complications according to the Clavien-Dindo classification. These findings suggest that albumin and CRP can serve as reliable biomarkers to predict complications, which is consistent with findings from other studies that have linked nutritional status and inflammatory markers to postoperative outcomes in cancer patients.^[25]^ We recommend that preoperative evaluations carefully monitor albumin levels as part of the preoperative risk assessment. Moreover, in patients with low albumin levels awaiting surgery, nutritional support may help reduce postoperative risk and improve overall recovery.

Several studies have highlighted the role of albumin in modulating cancer progression, where low serum albumin levels correlate with poor prognosis.^[26]^ The antioxidant properties of albumin, as well as its role in modulating immune responses and maintaining vascular integrity, may explain its importance in the context of ILP, where inflammation and tissue perfusion are pivotal.^[20]^ Given these findings, we believe that addressing preoperative nutritional deficiencies may help optimize patient outcomes.

While our study did not find a significant impact of body mass index on complications, toxicity, or mortality, it is important to note that the sample size may not have been large enough to detect subtle associations. Moreover, the absence of morbidly obese patients in our cohort and the fact that only 12% of patients were classified as obese may have limited the ability to draw definitive conclusions about the role of body mass index in ILP outcomes.

Our findings also emphasize the safety of combining ILP with resection procedures, as we did not observe any significant increase in complications, toxicity, or mortality when ILP was performed alongside surgical excisions. This supports the feasibility of performing ILP and resection concurrently under optimal conditions. Moreover, the number of lymph nodes removed did not appear to correlate with complications, which suggests that ILP does not significantly impact the risk of complications associated with lymphadenectomy.

The heterogeneous nature of our patient cohort, which included both melanoma and sarcoma cases, limited the ability to explore histological differences in the response to ILP. Given that ILP might have varying effectiveness depending on the tumor type and histological subtype, future studies should focus on stratifying patients based on these factors to better understand how tumor biology influences treatment outcomes. A personalized approach, based on histological subtypes, may help optimize ILP efficacy and minimize complications.

Although ILP achieved acceptable regional safety with low rates of severe regional toxicity, the overall mortality of our cohort remained high. This finding should be interpreted in the context of the advanced nature of the underlying malignancies rather than the ILP procedure itself. In our cohort, most deaths were attributable to metastatic disease rather than ILP-related complications, further supporting that long-term survival is predominantly driven by tumor biology rather than regional treatment alone.

One of the main limitations of our study is the relatively small sample size. A larger cohort would allow for more robust statistical analyses, enabling us to identify additional predictive markers of complications and toxicity. Moreover, the small number of early postoperative mortality events made it challenging to draw definitive conclusions about the factors influencing postoperative mortality. Future studies with larger sample sizes and more extensive follow-up are essential to refine the understanding of the factors that contribute to ILP-related complications.

In addition, the low number of patients experiencing severe toxicity limited our ability to identify statistically significant predictors of this outcome. As the application of ILP continues to grow, multicenter studies with larger patient populations would be invaluable in providing more conclusive data on the predictive factors for severe toxicity and mortality.

Finally, our study highlights the potential role of preoperative nutritional support in reducing postoperative risk after ILP. Given the importance of albumin and other biomarkers, future studies should explore the impact of nutritional prehabilitation on postoperative outcomes, particularly for patients at risk of nutritional deficiencies or those with compromised immune function.

## 5. Conclusion

ILP remains a valuable regional treatment option for carefully selected patients with advanced melanoma and ESTS when performed with appropriate care, particularly in the early postoperative period. Focusing on preoperative optimization of nutritional status and early postoperative monitoring may contribute to improved perioperative risk management. Our study emphasizes the need for personalized approaches to ILP, with a focus on biomarkers such as albumin and CRP to predict complications. Further research is necessary to refine these findings and explore additional factors influencing the success of ILP in patients with malignant melanoma and sarcoma.

## Author contributions

**Conceptualization:** Ezgi Altinsoy.

**Data curation:** Veysel Cem Özcan, Selim Tamam.

**Formal analysis:** Veysel Cem Özcan, Selim Tamam.

**Investigation:** Veysel Cem Özcan, Firat Tekeş, Selim Tamam.

**Project administration:** İsmail Can Tercan.

**Software:** İsmail Can Tercan.

**Methodology:** Firat Tekeş.

**Resources:** Firat Tekeş.

**Supervision:** Serdar Çulcu, Ali Ekrem Ünal.

**Writing – original draft:** Veysel Cem Özcan, Ezgi Altinsoy.

**Writing – review & editing:** Abdurrahman Başpinar.
